# Single-incision versus conventional laparoscopic appendectomy in adults: a systematic review and meta-analysis of randomized controlled trials

**DOI:** 10.1007/s13304-025-02112-5

**Published:** 2025-02-04

**Authors:** Konstantinos Kossenas, Riad Kouzeiha, Olga Moutzouri, Filippos Georgopoulos

**Affiliations:** 1 Department of Basic and Clinical Sciences, University of Nicosia Medical School, 21 Ilia Papakyriakou, 2414 Engomi, P.O. Box 24005, 1700 Nicosia, Cyprus; 2 Department of Medicine, Faculty of Medical Sciences, Lebanese University, Beirut, Lebanon; 3 Head of Gastroenterology and Hepatology at Al Zahra Hospital, Dubai, UAE

**Keywords:** Appendectomy, Single incision laparoscopic, Conventional laparoscopic surgery, Minimally invasive surgery, Experienced surgeons, Randomized clinical trials

## Abstract

Three-port (trocar) laparoscopic appendectomy is the standard treatment for acute appendicitis and previous studies have compared to single-incision approach, however, they often include both pediatric and adult patients and fail to account for surgeons’ experience, leading to variability in outcomes. This systematic review and meta-analysis aims to address these literature gaps by focusing on adult patients and controlling for surgeon expertise. We conducted a comprehensive search of randomized controlled trials comparing single-incision laparoscopic appendectomy (SILA) and conventional laparoscopic appendectomy (CLA) up to November 2024. We assessed the length of hospitalization, operative duration, postoperative complications, and surgical wound infections. Data were synthesized using random-effects models to account for variability among studies. The meta-analysis included four studies with a total of 408 patients, comprising 202 in the single-incision laparoscopic appendectomy (SILA) group and 206 in the conventional laparoscopic appendectomy (CLA) group. For the length of hospitalization, no statistically significant difference was observed, with a weighted mean difference (WMD) of 0.07 days (95% CI  − 0.32 to 0.47, *I*^2^ = 0%, *p* = 0.72). Similarly, the operative duration showed no significant difference, with a WMD of 4.49 min (95% CI  − 7.02 to 16.00, *I*^2^ = 89%, *p* = 0.44). The analysis of postoperative complications also revealed no significant difference between the groups, with an odds ratio (OR) of 1.32 (95% CI 0.69 to 2.51, *I*^2^ = 0%, *p* = 0.40). Surgical wound infections were found to be comparable, with an OR of 1.14 (95% CI 0.46 to 2.83, *I*^2^ = 0%, *p* = 0.78). Sensitivity analysis indicated that the results were statistically significant regarding operative duration when Kim et al. was excluded from the analysis. SILA and CLA yield comparable outcomes in terms of hospitalization length, operative duration, and complications, suggesting that both techniques are viable options for the management of acute appendicitis in adults. Further studies investigating overall cosmesis, patient satisfaction, and postoperative pain are warranted to optimize surgical approaches.

PROSPERO registration: CRD42024612596.

**Supplementary Information:**

The online version contains supplementary material available at 10.1007/s13304-025-02112-5.

## Introduction

Laparoscopic appendectomy is considered the preferred surgical treatment of acute appendicitis in most cases where expertise and equipment is available, due to the fact that it results in reduced postoperative pain, shorter hospital stays, and faster recovery compared to open appendectomy (OA) [1], especially because of its minimally invasive nature [2]. However, within the realm of laparoscopic techniques, a new approach has emerged: single-incision laparoscopic appendectomy (SILA). This method aims to further minimize the invasiveness of the procedure by utilizing a single incision, typically at the umbilicus, in contrast to the three or four incisions used in conventional laparoscopic appendectomy (CLA) [3].

While some studies suggest that SILA additionally offers potential benefits such as improved cosmetic outcomes, reduced hospital costs and stays, minimizing surgical incisions and enhanced patient experience, its widespread adoption has been tempered by concerns. These concerns include increased postoperative pain particularly upon exertion, the requirement of higher doses of analgesics, greater chance of complications, longer operative times compared to the conventional laparoscopic procedure, and the technically demanding nature of this surgical maneuver which requires specialized instruments and advanced surgical skills [4, 5].

Concurrently, other studies and trials have yielded results suggesting that SILA achieves comparable outcomes to CLA and highlighting a lack of significant differences between the two techniques in numerous domains [6].

Given the ongoing debate and the diverse findings from individual studies, a comprehensive systematic review and meta-analysis is necessary to provide a clearer understanding of the relative benefits and risks of SILA compared to CLA in adult patients. This systematic review aims to synthesize existing evidence from randomized controlled trials (RCTs) to evaluate the efficacy, safety, and postoperative outcomes of SILA versus CLA, when they are performed by experienced surgeons.

Surgeon's experience is a confounding factor in surgical studies because it can significantly influence patient outcomes through variability in technique, the learning curve associated with new procedures, and better patient selection and postoperative management. Experienced surgeons may achieve lower complication rates and better overall results due to their skill and familiarity with specific techniques, which can skew comparisons between surgical methods if not properly controlled. Failure to account for a surgeon's experience can lead to misleading conclusions about the safety and effectiveness of surgical interventions. We have observed that former systematic reviews and meta-analysis do not account for surgeon’s experience as a confounder.

By consolidating data from multiple studies, this meta-analysis seeks to offer more definitive conclusions that can inform clinical decision-making and surgical practice. The results of this review will have significant implications for surgeons considering the adoption of SILA and for patients exploring minimally invasive options for appendectomy. Ultimately, this review aims to determine whether SILA offers similar outcomes to CLA, when performed by experienced surgeons.

## Materials and methods

WE adhered to the recommended guidelines of the Cochrane collaboration [7] as well as the Preferred Reporting Items for Systematic Reviews and Meta-Analysis (PRISMA) guidelines [8] (supplementary Table 1). The methodology of this study was registered prospectively in PROSPERO with identification number CRD42024612596.

### Eligibility criteria, exclusion criteria, and PICOTT

We performed an extensive and systematic literature search up to the 7th of November 2024. The eligibility criteria for this review followed the PICOTT format (population, intervention, control, outcomes, type of studies, time of follow-up). The population studied (P) were adult patients, 18 years old or older, with any indication for appendectomy, who underwent (I) SILA and were compared (C) against CLA. Surgeries were performed by experienced surgeons. The outcomes (O) assessed were the length of hospitalization, operative duration, postoperative complications, and surgical wound infections. This review focuses on therapeutic interventions of only RCTs, directly comparing the two approaches (T) and the time of follow-up (T) was unrestricted. Exclusion criteria included studies that did not fit the PICOTT question, did not report any outcomes, or did not directly compare the two techniques.

### Database search

PubMed, Scopus, and Cochrane Library were used to retrieve relevant articles. The search term used was the following: (“Single-incision laparoscop*” OR “single-port laparoscop*” OR SILA) AND (“conventional laparoscop*” OR “standard laparoscop*” OR “multiport laparoscop*” OR “three-port laparoscop*”) AND (appendectomy OR appendicitis OR appendix OR “Appendicular abscess” OR “Appendicular mass” OR “Appendicular tumour” OR “complicated appendectomy” OR periappendiceal). At this stage of this review, we did not set any restrictions, i.e., age or surgeon’s experience. After we retrieved the studies, two authors (KK/OM) independently screened the titles and abstracts. The articles that were not excluded in title and abstract screening underwent full-text screening and were screened fully against the inclusion and exclusion criteria, by KK and OM, independently. Once the articles to be included in this review were identified, we cross-checked their reference list for any additional studies that could have been missed during the preliminary search. Conflicts were resolved by the supervisor (FG).

### Data extraction

Two authors, KK/RK, independently, performed data extraction, using two pre-deisgned Excel tables. The first one included study and patient baseline characteristics, such as the author of the study, country, number of patients undergoing each procedure, total number of patients, age, severity of appendicitis, surgical technique, and follow-up. The second table included the outcomes of interest. If fewer than ten studies were included in the meta-analyses, it was agreed that both reviewers would independently extract data from each study, followed by cross-checking to prevent errors during data transfer. No assumptions or simplifications were made during the data extraction process, as all information was directly obtained from the published studies without contacting the authors.

### Assessment of study quality and statistical analysis

To perform quality assessment of the included studies, we utilized the RoB 2 (Revised Cochrane risk-of-bias tool for randomized trials) at the study level. Quality assessment was performed by two reviewers (KK/RK) independently. For data analysis we utilized the odds ratios (OR), using the Mantel–Haenszel's formula, when the outcomes were dichotomous and the inverse variance mean differences when the outcomes were continuous. For both types of outcomes, we chose the random effects models as they consider the variability between studies as well as assume that the true effect sizes differ among the studies. Fixed-effects model was used as part of the sensitivity analysis. Moreover, a second sensitivity analysis (with excluding one study at the time-"leave one out") took place, to examine the extent that each study contributed to the observed variability. When studies reported their outcomes as median with ranges, they were converted to mean and standard deviation as described by Wan et al. [9]. Higgins I^2^ statistics was used to determine statistical heterogeneity, as described by the Cochrane Handbook [10]. Significant differences were considered when *p* value < 0.05. Funnel plots, to assess publication bias, were not utilized as we did not retrieve sufficient studies (10 or more). Cochrane Review Manager Tool (RevMan 5.4) software was used to conduct the meta-analysis [11].

## Results

### 3.1. Screening results

**Fig. 1:**
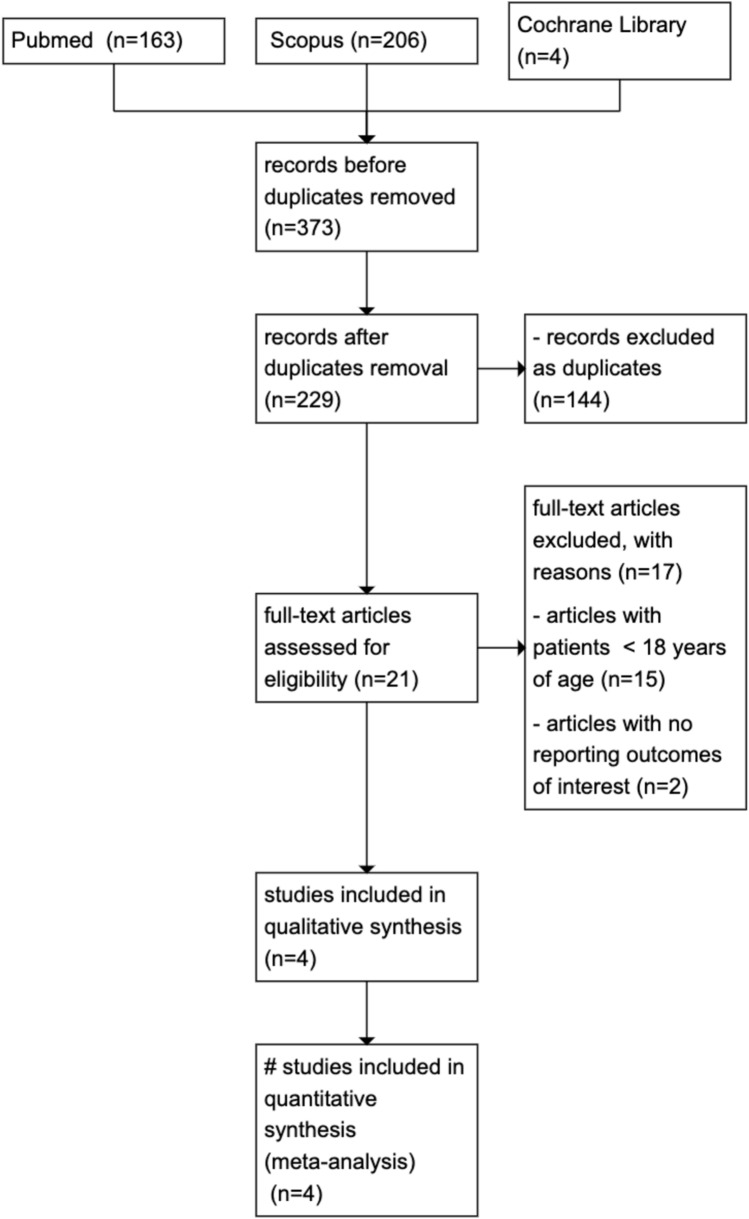
PRISMA flowchart

A total of 373 articles were retrieved from three databases: 163 from PubMed, 206 from Scopus, and 4 from the Cochrane Library. Using Rayyan Software, we manually identified and removed 144 duplicate records. This left 229 articles for title and abstract screening. Of these, 21 articles were selected for full-text review, and data were ultimately extracted from four studies. The screening process is illustrated in the PRISMA flowchart (Fig. 1).

### 3.2. Study characteristics and patients’ demographics

A total of 195 patients were studied by Teoh et al. [15] in Hong Kong, focusing on generalized peritonitis, while Kim et al. [13] included 98 patients in Korea with suppurative, gangrenous, or perforated appendicitis. Carter et al. [12] conducted their research in the USA with 75 patients, examining various severities of appendicitis, and Park et al. [14], also from Korea, studied 40 patients with perforated appendicitis. The surgical techniques varied, with Carter et al. [12] using a 12 mm umbilical incision and two 5 mm ports; Kim et al. [13] employing a 10 mm umbilical incision with two 5 mm trocars; Park et al. [14] utilizing two 5 mm trocars and one 10 mm trocar; and Teoh et al. [15] using two 5 mm ports. Follow-up periods ranged from 1 to 6 weeks across the studies (Table 1).

**Table 1:** Study characteristics

Author	Country	Number of patients			Age	Severity of appendicitis	Surgical technique	Follow up
		SILA	CLA	TOTAL				
Carter et al. [12]	USA	37	38	75	> 23	inflammation, mass, periappendicecal abscess, or diffuse peritonitis	The surgical approach involves a 12 mm umbilical incision for entry, supplemented by two 5 mm ports: one in the left lower quadrant and another in the suprapubic midline. A linear stapler or looped suture is used as a cutting-and-sealing device during the procedure	2–3 weeks
Kim et al. [13]	Korea	47	51	98	26–56	Suppurative or gangrenous or perforates appendicitis	a 10 mm umbilical incision and two additional 5 mm trocars in the left lower quadrant and suprapubic region. The appendix's base is ligated with Endoloops or an Endo-GIA stapler	6 weeks
Park et al. [14]	Korea	20	20	40	> 25	Perforated appendicitis or periappendiceal abscess	two 5 mm trocars and one 10 mm trocar. An Endoloop is employed for closure, along with scalpels or endoclips for dissection and securing tissues	1 weeks
Teoh et al. [15]	Hong Kong	98	97	195	18–75	Generalized peritonitis or abscess or abdominal mass	wo 5 mm ports placed in the left and right lower quadrants. An Endoloop is utilized for closure, alongside ultrasonic shears for dissection and tissue cutting	14 days

SILA: single incision laparoscopic appendectomy, CLA: conventional laparoscopic appendectomy

### 3.3. Operative outcomes

The length of hospitalization for SILA ranged from approximately 1.4 to 3.53 days, while CLA ranged from 1.6 to 3.2 days. Operative durations for SILA were generally longer, with averages around 52–63.5 min compared to 38–63 min for CLA. Postoperative complications were observed in both groups, with SILA reporting between 2 and 14 complications and CLA reporting 4 and 9, indicating that SILA may have a higher complication rate in some studies. Surgical wound infections were low for both techniques, with SILA ranging from 0 to 8 infections and CLA from 0 to 5 (Table 2).

**Table 2:** Surgical outcomes of SILA vs CLA

Authors	Length of hospitalization		Operative duration		Post-operative complication s		Surgical wound infections	
	SILA	CLA	SILA	CLA	SILA	CLA	SILA	CLA
Carter et al. [12]	1.4 ± 0.8	1.6 ± 1.8	54 ± 17	38 ± 12	5	4	0	0
Kim et al. [13]	2.89 ± 1.56	2.71 ± 1.89	52.2 ± 19.4	63 ± 21	3	4	2	4
Park et al. [14]	N/a	N/a	63.5 ± 13.2	54 ± 12.5	2	2	1	1
Teoh et al. [15]	3.53 ± 2.92	3.2 ± 2.36	63 ± 27.2	60.2 ± 31.7	14	9	8	5

SILA: single incision laparoscopic appendectomy, CLA: conventional laparoscopic appendectomy

### 3.4. Study quality

The assessment of studies using the ROB 2 tool reveals varying levels of risk of bias across the five domains (D1 to D5) for each author. Carter et al. [12] demonstrated low risk in all domains, indicating a robust study design and execution. Kim et al. [13] showed a potential concern in D2 regarding deviations from intended interventions, leading to an overall moderate risk of bias. Park et al. [14] had an unclear risk in D1, suggesting uncertainty in the randomization process, but overall maintained a moderate risk across other domains. Teoh et al. [15] scored favorably with low risk in all domains, reflecting strong methodological rigor. Overall, the findings indicate that while some studies exhibit strong risk management, others present potential weaknesses that could impact the validity of their conclusions (Table 3).

**Table 3:**
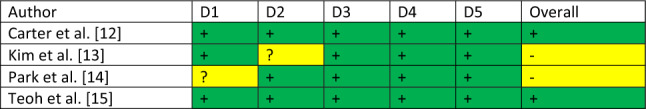
Assessment of study quality using RoB 2

In the ROB 2 (Risk of Bias 2) tool, **D1 to D5** represent five key domains used to assess the risk of bias in randomized controlled trials. **D1** evaluates the adequacy of the randomization process to prevent selection bias, ensuring that participants are allocated to intervention groups fairly. **D2** examines whether participants received the intended interventions as assigned, assessing any deviations that may affect the outcomes. **D3** focuses on the handling of missing outcome data, considering the impact of any missing information on the study’s conclusions. **D4** looks at the measurement of outcomes, specifically whether outcome assessors were blinded to group allocations and if the methods used were reliable and consistent. Lastly, **D5** assesses the risk of bias in the selection of reported results, ensuring that all pre-specified outcomes were reported transparently, without selective omission of unfavorable findings. Together, these domains provide a comprehensive framework for evaluating the overall risk of bias in a study

## Meta-analysis

**Fig. 2:**
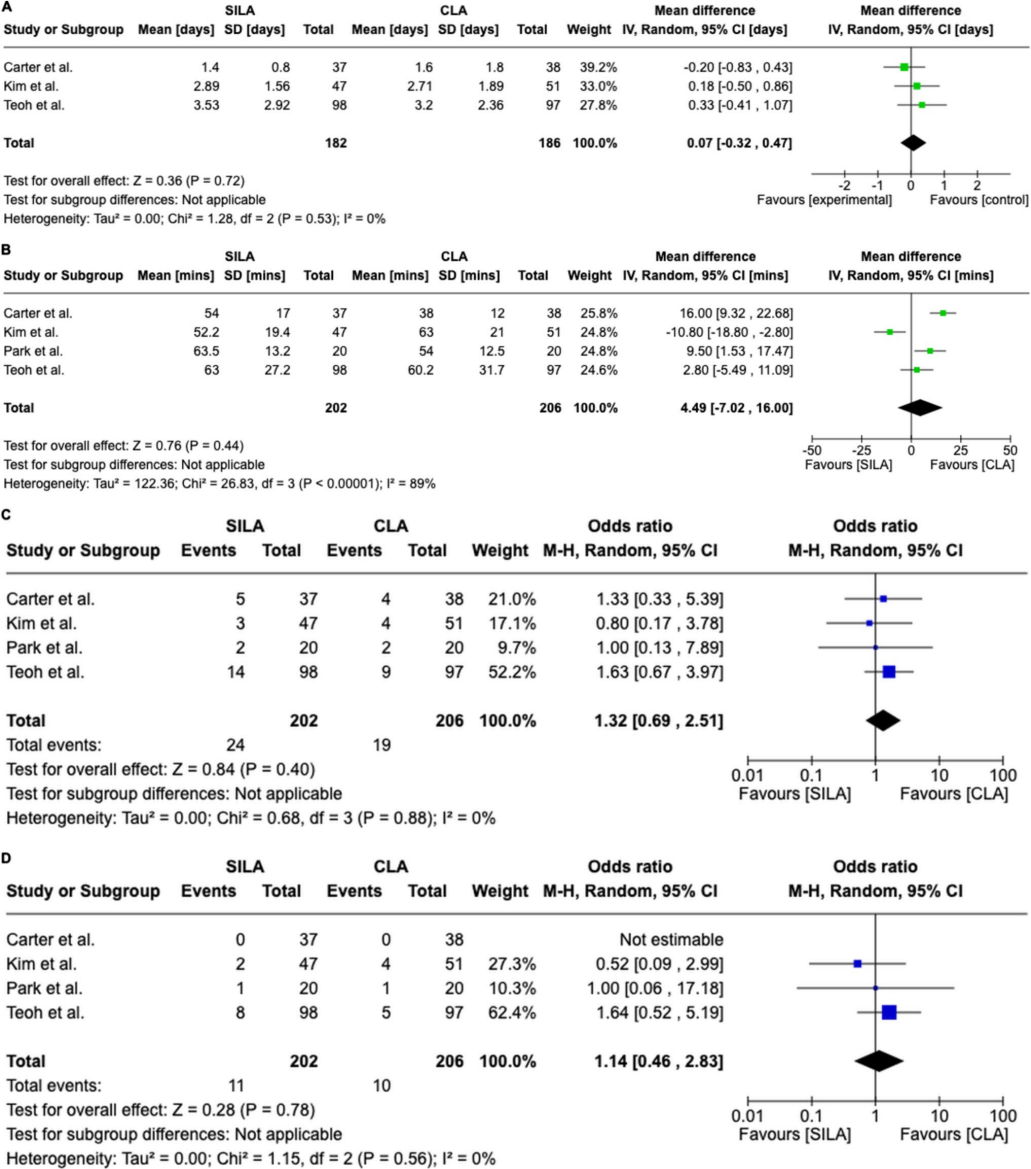
Meta-analysis; A: length of hospitalization, B: operative duration, C: postoperative complications, D: surgical wound infections

Length of hospitalization, operative duration, postoperative complications, and surgical wound infections were meta-analyzed (Fig. 2).

### Length of hospitalization

Three studies with a total of 368 patients, 182 in SILA and 186 in CLA, investigated the length of hospitalization and showed a non-statistically significant difference between the two approaches [12, 13, 15]. WMD = 0.07 days [95%CI  − 0.32, 0.47], I2 = 0%, *p* = 0.72.

### Operative duration

Four studies with a total of 408 patients, 202 in SILA and 206 in CLA, investigated the operative duration and showed a non-statistically significant difference between the two approaches [12–15]. WMD = 4.49 min [95%CI  − 7.02, 16.00], I2 = 89%, *p* = 0.44.

### Postoperative complications

Four studies with a total of 408 patients, 202 in SILA and 206 in CLA, investigated the postoperative complications and showed a non-statistically significant difference between the two approaches [12–15]. OR = 1.32 [95%CI 0.69, 2.51], I2 = 0%, *p* = 0.40.

### Surgical wound infections

Four studies with a total of 408 patients, 202 in SILA and 206 in CLA, investigated the surgical wound infections and showed a non-statistically significant difference between the two approaches [12–15]. OR = 1 1.14 [95%CI 0.46, 2.83], I2 = 0%, *p* = 0.78.

### Sensitivity analysis

We performed a sensitivity analysis by excluding one study at a time (“leave one out”) (Table 4) and one with fixed-effects models (Table 5).

**Table 4:** Sensitivity analysis by excluding each study for each outcome

Study excluded	WMD/OR [95%CI]	*I*^2^	*p* value
Length of hospitalization			
Carter et al. [12]	0.25 [ − 0.26, 0.75]	0	0.33
Kim et al. [13]	0.03 [ − 0.49, 0.54]	12	0.92
Teoh et al. [15]	−0.03 [ − 0.49, 0.44]	0	0.91
Operative Duration			
Carter et al. [12]	0.49 [ − 11.34, 12.33]	84	0.93
Kim et al. [13]	9.75 [2.14, 17.36]	67	0.01
Park et al. [14]	2.78 [ − 12.99, 18.55]	92	0.73
Teoh et al. [15]	5.00 [ − 10.76, 20.76]	92	0.53
Post-operative complications			
Carter et al. [12]	1.32 [0.64, 2.71]	0	0.46
Kim et al. [13]	1.46 [0.72, 2.96]	0	0.29
Park et al. [14]	1.36 [0.69, 2.67]	0	0.37
Teoh et al. [15]	1.05 [0.41, 2.65]	0	0.92
Surgical Wound infections			
Kim et al. [13]	1.53 [0.52, 4.45]	0	0.44
Park et al. [14]	1.12 [0.39, 3.22]	13	0.83
Teoh et al. [15]	0.62 [0.14, 2.76]	0	0.53

WMD: weighted mean difference, OR: odds ratio, CI confidence interval

**Table 5:** Sensitivity analysis with Fixed effects model

Outcome	WMD/OR [95%CI]	*I*^2^	*p* value
Length of Hospitalization	0.07 [ − 0.32, 0.47]	0	0.72
Operative Duration	5.57 [1.74, 9.39]	84	0.04
Post-operative complications	1.32 [0.70, 2.49]	0	0.39
Surgical ound infections	1.13 [0.47, 2.73]	0	0.79

WMD: weighted mean difference, OR: odds ratio, CI confidence interval

The results were statistically significant with regard to operative duration when Kim et al.’s study was excluded [13], in the fixed-effect model.

## Discussion

In this systematic review and meta-analysis, we evaluated the outcomes associated with SILA compared to CLA. The findings revealed no significant differences in key postoperative metrics, including the length of hospitalization, operative duration, postoperative complications, and surgical wound infections between the two techniques. Notably, while SILA is often promoted for its potential cosmesis advantages, our results suggest that its efficacy and safety are comparable to those of CLA when performed by experienced surgeons. Previous studies have frequently included a heterogeneous patient population, encompassing both pediatric and adult patients, and often failed to account for critical factors such as age and the experience level of the surgeons involved. This oversight may contribute to variability in outcomes and limit the generalizability of the findings. By focusing exclusively on adult patients operated on by experienced surgeons, our study aimed to provide clearer insights into the relative benefits and risks of these surgical approaches, thereby addressing the existing literature gap.

Other studies have previously investigated the two approaches. In the study conducted by Duza et al. [16], which included patients over the age of 14 years, CLA was associated with shorter preoperative and intraoperative times compared to SILA, which showed reduced postoperative and reinsertion times. However, there were no significant differences in postoperative complications or cosmetic satisfaction between the two approaches. In comparison, our review found SILA and CLA produced similar outcomes in operative duration and complications, suggesting that the differences in timing noted by Duza et al. [16] may not be as pronounced in an adult population.

Golebiewski et al. [17] evaluated SILA against three-port laparoscopic appendectomy in a pediatric cohort, finding that although the operative time for three-port laparoscopic appendectomy was shorter, patients undergoing SPLA reported greater postoperative pain and increased levels of inflammatory markers, with no significant differences in hospital stay or complications. Our review indicated that SILA and CLA yielded comparable results regarding hospitalization length and postoperative complications, suggesting that the increased pain associated with SPLA in Golebiewski et al.'s study needs to be investigated in future adult populations [17].

In a diverse patient population aged 7–71 years, Kang et al. [18] reported a conversion rate of 11.1% to MPLS and found that SPLS was associated with longer recovery times and increased analgesic use. In contrast, our findings suggested that SILA and CLA had comparable outcomes in hospitalization length, operative duration, and complications, indicating that the higher recovery time noted in Kang et al.'s study may not be a consistent finding across different population groups [18].

Lee et al. [19] found no significant differences in overall complication rates or cosmetic satisfaction between SPLA and CLA in patients over 16 years, aligning with our results that suggest SILA and CLA yield similar outcomes concerning complications. In the study by Pan et al. [20], TSILA showed comparable operative times to the classic method and higher cosmetic satisfaction, but our review indicated that while SILA and CLA produced similar results, SILA’s advantages were less evident when factoring in the operational efficiency of CLA. However, the cosmesis effects needs to be further evaluated. The SCARLESS study group [21] reported that patients undergoing SPILS experienced better body image and cosmetic satisfaction, although it was technically more demanding. In contrast, our review highlighted that both SILA and CLA had similar outcomes, suggesting that while cosmetic benefits may be present with SILA in other studies, they did not translate into significant clinical differences when compared to CLA.

Lastly, Cirochi et al. [22] found that SILA had a longer operative time, but offered similar outcomes to CLA across all age groups. However, our review showed a non-significant difference in the operative duration. This consistency underscores the need for further research into postoperative pain and cosmetic outcomes, as our review indicated that both techniques are viable options for managing acute appendicitis without significant differences in clinical outcomes.

The findings from this systematic review and meta-analysis offer significant implications for health policymakers, healthcare providers, and patients regarding the management of acute appendicitis. For health policymakers, the comparable outcomes between SILA and CLA indicate that both techniques should be integrated into clinical guidelines, allowing flexibility in surgical approaches based on surgeon expertise and patient preferences. This could optimize resource allocation and improve overall surgical care. Surgeons are reminded of the critical role that experience plays in influencing surgical outcomes, as our study demonstrates that both techniques yield similar results in terms of hospitalization length, operative duration, and complications. This reinforces the notion that surgeons can confidently choose either approach based on their proficiency and the individual needs of patients, ultimately enhancing training programs that emphasize skill development in both methods. From the patient perspective, understanding that SILA and CLA are equally viable options can empower them to make informed decisions about their treatment. This knowledge may alleviate concerns associated with opting for a single-incision technique, particularly given its potential cosmetic advantages.

Surgeons adopting SILA and patients willing to undergo this type of surgery need to be aware of trocar-site hernia. A study by Antoniou et al. [23], which examined randomized clinical trials and compared their risk of trocar-site hernia between single-incision laparoscopic surgery (SILS) and conventional laparoscopic surgery, concluded that SILS had a significantly higher risk of trocar-site hernia compared to conventional laparoscopic surgery (odds ratio = 2.37, *p* = 0.008). Trial sequential analysis confirmed conclusive evidence of increased trocar-site hernia risk with SILS.

With the rise of minimally invasive surgery in general surgery [24–27], further research is essential to investigate patient-reported outcomes such as postoperative pain and satisfaction, as these factors significantly influence the overall patient experience and recovery trajectory. Understanding the nuances of postoperative pain levels between SILA and CLA will help clinicians better prepare patients for what to expect following surgery, ultimately leading to improved pain management strategies. Additionally, capturing patient satisfaction data can provide valuable insights into how different surgical approaches align with patient preferences and perceptions of care quality. These outcomes can be measured using standardized tools that assess not only the severity of pain, but also the patient's emotional and psychological well-being throughout the recovery period. Enhanced focus on these patient-reported outcomes can facilitate shared decision-making processes, allowing patients to engage actively in their treatment choices. By integrating findings from such research into clinical practice, healthcare providers can better tailor their approaches to align with individual patient values and preferences, fostering a more patient-centered care environment. Ultimately, a comprehensive understanding of postoperative experiences will contribute to improved surgical outcomes, heightened patient satisfaction, and more effective communication between patients and healthcare professionals, promoting an overall enhancement in the quality of surgical care for acute appendicitis.

This systematic review and meta-analysis has several limitations that should be acknowledged. First, the number of studies included in the analysis was limited, which may affect the generalizability of the findings. The studies analyzed had variability in their designs, surgical techniques, and definitions of outcomes, leading to potential heterogeneity in the data. The follow-up durations varied significantly among studies, potentially impacting the assessment of long-term complications and patient satisfaction. Furthermore, the studies included in the review did not extensively evaluate important factors such as postoperative pain and cosmesis, which are critical in assessing the overall success of surgical interventions. Finally, the exclusion of non-English language studies and unpublished data may have introduced publication bias, limiting the comprehensiveness of the evidence presented. Future research should aim to address these limitations by including a larger pool of studies with consistent methodologies and more rigorous reporting on surgeon experience and patient-reported outcomes.

## Conclusion

In conclusion, this systematic review and meta-analysis provide valuable insights into the comparative effectiveness of single-incision laparoscopic appendectomy (SILA) versus conventional laparoscopic appendectomy (CLA) in adults, emphasizing the importance of surgeon experience in achieving optimal outcomes. Our findings demonstrate that both SILA and CLA yield similar results concerning length of hospitalization, operative duration, and rates of postoperative complications and surgical wound infections. This suggests that both techniques are viable options for managing acute appendicitis when performed by experienced surgeons. However, to enhance the decision-making process for both patients and healthcare providers, further research is warranted to explore patient-reported outcomes, such as postoperative pain and satisfaction, which are critical for understanding the overall impact of these surgical approaches on patient quality of life. By addressing these factors, we can improve shared decision-making processes and ensure that surgical interventions align with patient preferences and expectations. As surgical techniques continue to evolve, it is essential to integrate findings from ongoing research into clinical practice, ultimately enhancing the quality of care provided to patients undergoing appendectomy.

## Supplementary Information

Below is the link to the electronic supplementary material.

Supplementary file 1 (DOCX 35 KB)

## Data Availability

Not applicable.

## References

[1] Sauerland S, Jaschinski T, Neugebauer EA (2010) Laparoscopic versus open surgery for suspected appendicitis. Cochrane Database Syst Rev. 10.1002/14651858.CD001546.pub3 20927725 10.1002/14651858.CD001546.pub3

[2] Gray KD, Burshtein JG, Obeid L, Moore MD, Dakin G, Pomp A, Afaneh C (2018) Laparoscopic appendectomy: minimally invasive surgery training improves outcomes in basic laparoscopic procedures. World J Surg 42:1706–1713. 10.1007/s00268-017-4374-z 29143092 10.1007/s00268-017-4374-z

[3] Joliat GR, Uldry E, Demartines N, Schäfer M (2014) Single-incision versus conventional laparoscopic appendectomy: a case-match study. SAGE Open Med 2:2050312114524195. 10.1177/2050312114524195 26770712 10.1177/2050312114524195 PMC4607210

[4] Rao PP, Rao PP, Bhagwat S (2011) Single-incision laparoscopic surgery - current status and controversies. J Minim Access Surg 7:6–16. 10.4103/0972-9941.72360 21197236 10.4103/0972-9941.72360 PMC3002008

[5] Chen Y, Fan Z, Zhang X, Fu X, Li J, Yuan J, Guo S (2023) A brief overview of single-port laparoscopic appendectomy as an optimal surgical procedure for patients with acute appendicitis: still a long way to go. J Int Med Res 51:3000605231183781. 10.1177/03000605231183781 37466195 10.1177/03000605231183781 PMC10363874

[6] Clerveus M, Morandeira-Rivas A, Moreno-Sanz C, Herrero-Bogajo ML, Picazo-Yeste JS, Tadeo-Ruiz G (2014) Systematic review and meta-analysis of randomized controlled trials comparing single incision versus conventional laparoscopic appendectomy. World J Surg 38:1937–1946. 10.1007/s00268-014-2535-x 24682257 10.1007/s00268-014-2535-x

[7] Higgins JPT, Green S (2008) Cochrane handbook for systematic reviews of interventions. Cochrane Book Series. 10.1002/9780470712184

[8] Moher D, Liberati A, Tetzlaff J, Altman DG (2009) Preferred reporting items for systematic reviews and meta-analyses: the PRISMA statement. BMJ. 10.1136/bmj.b2535 21603045 PMC3090117

[9] Wan X, Wang W, Liu J, Tong T (2014) Estimating the sample mean and standard deviation from the sample size, median, range and/or interquartile range. BMC Med Res Methodol 14:135. 10.1186/1471-2288-14-135 25524443 10.1186/1471-2288-14-135 PMC4383202

[10] Schünemann HJ, Higgins JPT, Vist GE, Glasziou P, Akl EA, Skoetz N, Guyatt GH (on behalf of the Cochrane GRADEing Methods Group) (n.d.) GRADE guidelines: 10. Assessing the certainty of evidence in a synthesis of findings. https://training.cochrane.org/handbook/current/chapter-14

[11] Review Manager (RevMan) (n.d.) Version (online). The Cochrane Collaboration. Available at revman.cochrane.org

[12] Carter JT, Kaplan JA, Nguyen JN, Lin MY, Rogers SJ, Harris HW (2014) A prospective, randomized controlled trial of single-incision laparoscopic vs conventional 3-port laparoscopic appendectomy for treatment of acute appendicitis. J Am Coll Surg 218:950–959. 10.1016/j.jamcollsurg.2013.12.052 24684867 10.1016/j.jamcollsurg.2013.12.052

[13] Kim KE, Cho IS, Bae SU, Jeong WK, Kim HJ, Baek SK (2023) A prospective randomized controlled study comparing patient-reported scar evaluation of single-port versus multiport laparoscopic appendectomy for acute appendicitis. J Minim Invasive Surg 26:55–63. 10.7602/jmis.2023.26.2.55 37347098 10.7602/jmis.2023.26.2.55 PMC10280108

[14] Park JH, Hyun KH, Park CH, Choi SY, Choi WH, Kim DJ (2010) Laparoscopic vs transumbilical single-port laparoscopic appendectomy; results of prospective randomized trial. J Korean Surg Soc 78:213–218. 10.4174/jkss.2010.78.4.213

[15] Teoh AY, Chiu PW, Wong TC et al (2012) A double-blinded randomized controlled trial of laparoendoscopic single-site access versus conventional 3-port appendectomy. Ann Surg 256:909–914. 10.1097/SLA.0b013e3182765fcf 23154391 10.1097/SLA.0b013e3182765fcf

[16] Duza G, Davrieux CF, Palermo M et al (2019) Conventional laparoscopic appendectomy versus single-port laparoscopic appendectomy, a multicenter randomized control trial: a feasible and safe alternative to standard laparoscopy. J Laparoendosc Adv Surg Tech A 29:1577–1584. 10.1089/lap.2019.0460 31613689 10.1089/lap.2019.0460

[17] Golebiewski A, Anzelewicz S, Wiejek A, Lubacka D, Czauderna P (2019) A prospective randomized controlled trial of single-port and three-port laparoscopic appendectomy in children. J Laparoendosc Adv Surg Tech A 29:703–709. 10.1089/lap.2018.0560 30945979 10.1089/lap.2018.0560

[18] Kang BM, Choi SI, Kim BS, Lee SH (2018) Single-port laparoscopic surgery in uncomplicated acute appendicitis: a randomized controlled trial. Surg Endosc 32:3131–3137. 10.1007/s00464-018-6028-0 29340826 10.1007/s00464-018-6028-0

[19] Lee WS, Choi ST, Lee JN et al (2013) Single-port laparoscopic appendectomy versus conventional laparoscopic appendectomy: a prospective randomized controlled study. Ann Surg 257:214–218. 10.1097/SLA.0b013e318273bde4 23241869 10.1097/SLA.0b013e318273bde4

[20] Pan Z, Jiang XH, Zhou JH, Ji ZL (2013) Transumbilical single-incision laparoscopic appendectomy using conventional instruments: the single working channel technique. Surg Laparosc Endosc Percutan Tech 23:208–211. 10.1097/SLE.0b013e3182827f5d 23579520 10.1097/SLE.0b013e3182827f5d

[21] SCARLESS Study Group, Ahmed I, Cook JA et al (2015) Single port/incision laparoscopic surgery compared with standard three-port laparoscopic surgery for appendicectomy: a randomized controlled trial. Surg Endosc 29:77–85. 10.1007/s00464-014-3416-y 25270609 10.1007/s00464-014-3416-y PMC4293491

[22] Cirocchi R, Cianci MC, Amato L et al (2024) Laparoscopic appendectomy with single port vs conventional access: systematic review and meta-analysis of randomized clinical trials. Surg Endosc 38:1667–1684. 10.1007/s00464-023-10659-w 38332174 10.1007/s00464-023-10659-w PMC10978699

[23] Antoniou SA, García-Alamino JM, Hajibandeh S et al (2018) Single-incision surgery trocar-site hernia: an updated systematic review meta-analysis with trial sequential analysis by the minimally invasive surgery synthesis of interventions outcomes network (MISSION). Surg Endosc 32:14–23. 10.1007/s00464-017-5717-4 28726142 10.1007/s00464-017-5717-4

[24] Kossenas K, Moutzouri O, Georgopoulos F (2024) Robotic vs laparoscopic distal gastrectomy with Billroth I and II reconstruction: a systematic review and meta-analysis. J Robot Surg 19:30. 10.1007/s11701-024-02193-1 39699804 10.1007/s11701-024-02193-1

[25] Kossenas K, Kalomoiris D, Georgopoulos F (2024) Single-port robotic versus single-incision laparoscopic cholecystectomy in patients with BMI ≥25 kg/m^2^: a systematic review and meta-analysis. J Robot Surg 19:2. 10.1007/s11701-024-02167-3 39549130 10.1007/s11701-024-02167-3

[26] Kossenas K, Karamatzanis I, Moutzouri O et al (2024) Precision versus practicality: a comprehensive analysis of robotic right colectomy versus laparoscopic right colectomy, future directions, biases, research gaps, and their implications. Cureus 16:e52904. 10.7759/cureus.52904 38406010 10.7759/cureus.52904 PMC10892367

[27] Kossenas K, Georgopoulos F (2023) The evolving surgical landscape: a comprehensive review of robotic versus laparoscopic gastrectomy for the treatment of gastric cancer. Cureus 15:e49780. 10.7759/cureus.49780 38161532 10.7759/cureus.49780 PMC10757755

